# Assessing Rodent Cardiac Function *in vivo* Using Hemodynamic Pressure-Volume Loops

**DOI:** 10.3389/fphys.2021.751326

**Published:** 2022-06-23

**Authors:** Daniela Miranda-Silva, Vasco Sequeira, André P. Lourenço, Inês Falcão-Pires

**Affiliations:** ^1^UnIC@RISE, Department of Surgery and Physiology, Faculty of Medicine, University of Porto, Porto, Portugal; ^2^Department of Translational Science, DZHI, Universitätsklinikum Würzburg, Würzburg, Germany

**Keywords:** heart failure, P-V loop, *in vivo* cardiac function, hemodynamics, conductance catheter

## Abstract

Heart failure (HF) triggered by cardiovascular and non-cardiovascular diseases is a leading cause of death worldwide and translational research is urgently needed to better understand the mechanisms of the failing heart. For this purpose, rodent models of heart disease combined with *in vivo* cardiac functional assessment have provided valuable insights into the physiological significance of a given genetic or pharmacological modification. In small animals, cardiac function and structure can be evaluated by methods such as echocardiography, telemetry or hemodynamics using conductance catheters. Indeed, hemodynamic analysis of pressure-volume loops (PV-loops) has become the gold standard methodology to study *in vivo* cardiac function in detail. This method provides simultaneous measurement of both pressure and volume signals from rodents intact beating hearts. On the one hand, PV-loop analysis has deeply expanded the knowledge on molecular cardiac physiology by allowing establishing important functional correlations. On the other hand, these measurements allow dissecting the cardiovascular functional impact of certain therapeutic interventions or specific signaling pathways using transgenic models of disease. However, a detailed assessment of cardiac function and structure *in vivo* still warrants proper standardization and optimization to boost the progress of HF research. With increasing concerns over data accuracy and reproducibility, guidelines and best practices for cardiac physiology measurements in experimental settings are needed. This article aims to review the best practices for carrying out cardiac hemodynamic assessment using PV-loops *in vivo* in rodents intact beating hearts, also providing an overview of its advantages, disadvantages and applications in cardiovascular research.

## Pressure-Volume Loops: Concept and Theory

In 1628, William Harvey described the complete systemic circulation, introducing the concept of hemodynamic. Throughout the centuries several authors, including Poiseuille, Frank, Starling, Wiggers, and Westerhof contributed to the understanding of blood pressure flow and dynamics. During the twentieth century, the golden era of hemodynamic evaluation, novel methods to accurate measured ventricular systolic and diastolic function were developed, including PV-loop analysis (Patil et al., 2017).

The initial description of ventricular function using PV relationships was done by Suga and Sagawa (1974) 40 years ago. Baan et al. (1984) designed a conductance catheter able to continuously acquire simultaneous pressure and volume data in large animals (Wei et al., 2014). The subsequent development of tools to genetically manipulate cardiovascular signaling pathways in rodents drove the miniaturization of sensors and techniques to assess cardiac function using PV-loops. Indeed, Kass’ laboratory firstly used PV catheters in small rodents to elucidate the hemodynamic implications of transgenic models of cardiovascular disease (Pacher et al., 2004; Tam et al., 2006; Shioura et al., 2007). PV catheters allow obtaining a continuous register of PV loops, under steady-state conditions or during transient preload reduction. Therefore, despite its invasiveness, hemodynamics is the most reliable available methodology to assess ventricular function independently of loading conditions while quantifying ventricular load and the interaction with vasculature (Pacher et al., 2008).

## Best Practices for Pressure-Volume-Loop Experimental and Surgical Preparation

**FIGURE 1:**
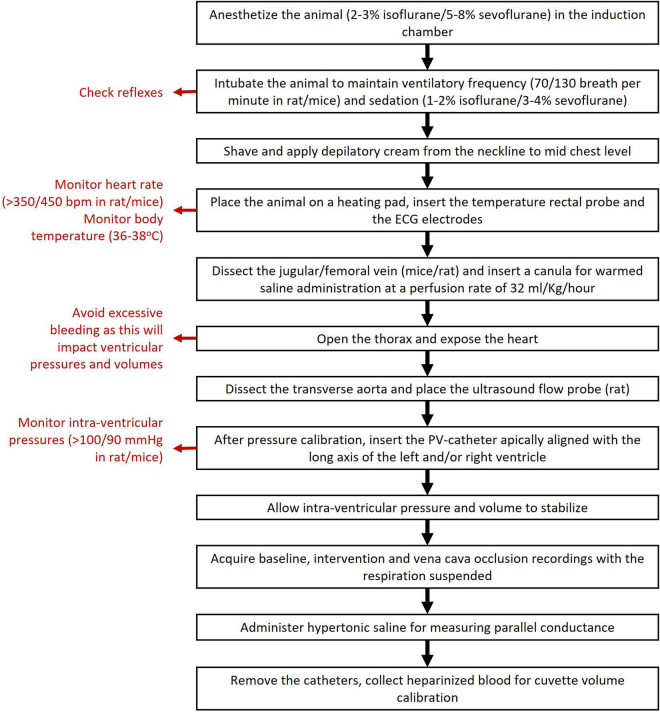
Main steps and recommendations (in red) for PV-loops experimentation.

To obtain high quality and reproducible PV-loop data, a trained operator is mandatory. Also, effective monitoring and tight control of vital parameters (temperature, cardiac output, and oxygen blood levels) are crucial during hemodynamic assessment using PV-loops. A flow chart with the main steps of the protocol is depicted in Figure 1.

The surgery table surface and all the material needed for the procedure should be wiped with a disinfectant before and after use. Aseptic techniques are particularly important if the hemodynamic evaluation is not a terminal procedure.

### Anesthesia

Acute procedures in anesthetized rodents are easier and more often used. In such cases, anesthesia should be carefully selected, considering that might vary significantly with age, strain (Ari et al., 2018), liver/lung function, obesity, and other diseases, such as HF and sepsis (Zacchigna et al., 2021). The most important considerations are that the selected anesthetic (1) provides an appropriate depth and length of anesthesia, (2) does not compromise important hemodynamic parameters assessment for the selected protocol or animal model, and (3) is safe both for the animal and the person administering anesthesia. For instance, ether, pentobarbital sodium and urethane influence mean arterial pressure, cardiac output, heart rate and total peripheral resistance, while the commonly used ketamine:xylazine (100:10 mg/Kg) injection has an overall cardio-depressant effect. Also, barbiturates should be avoided as these alter the animal’s ability to maintain core temperature. Thus, halogenated gasses (isoflurane or sevoflurane) are now preferred due to their easy titration, fast reversibility, and most importantly, minimal cardiovascular depression (Zacchigna et al., 2021). For a detailed overview of the advantages and limitations of the most common anesthetics used during hemodynamic evaluation in rodents, see Janssen et al. (2004), Obst et al. (2004), Lindsey et al. (2018).

### Ventilation

Immediately after intubation, the endotracheal tube is connected to the ventilator. In the case of using anesthetic gasses, the mixture is adjusted (2–3% isoflurane or 5–8% sevoflurane for induction followed by a reduction to 1–2% isoflurane or 3–4% sevoflurane). The flow of oxygen should exceed minute ventilation in non-rebreathing systems. The calculation of the ventilatory settings depend on the animal weight. If the ventilator is set to work in volume mode, the settings are calculated as follows:


$$\mathrm{Tidal}\,\mathrm{volume}\,\left(\mathrm{mL}\right)=6.2\times \mathrm{weight}\,{\left(\mathrm{Kg}\right)}^{1.01}$$

$$\mathrm{Respiratory}\,\mathrm{rate}\,\left(\mathrm{RR}/\min\right)=53.5\times \mathrm{weight}\,{\left(\mathrm{Kg}\right)}^{-0.26}$$

### Animal Preparation and Positioning

Nevertheless, a ventilator working in pressure mode will allow less lung trauma. The peak inspiratory pressure (PIP) can go up to 25 cm H20, depending on the respiratory rate or the physiological/pathologic condition. Ideally, an adequate ventilation should monitor O_2_ saturation and end-tidal CO_2_ with oximetry and capnography, respectively.

After intubation and induction of anesthesia, the toe of the animal should be pinched to assure that the animal is fully anesthetized. The hair from the animal’s chest should be removed with clippers or depilatory cream and the skin cleaned with disinfectant. In rats, the correct placement of the animal is usually at partial lateral-right decubitus, and the inferior left paw may cross the inferior right paw, helping in the animal positioning. When the femoral vein is used to administer fluids (pre-warmed saline solution) the correct positioning of the animal is done only after the catheterization. Mice are placed in a supine position and the paws taped to the heating pad. Usually, the jugular vein is the most frequently used route to administer fluids in mice.

### Temperature Control and Fluid Administration

It is extremely important to provide fluid support to maintain normal “blood volume” during all measurements (Zuurbier et al., 2002). To compensate for blood or fluid losses, the protocol should involve fluid administration of a pre-warmed saline solution (or any other crystalloid solution such as Ringer’s lactate) by intravenous perfusion, which can also be used as a vehicle for drug administration. Fluid administration is usually done with a syringe pump at a rate of 32 mL.Kg.hour. Bleeding should be minimized throughout the procedure and blood loss can be replaced 4/1 by crystalloid solutions. Volume infusion should be titrated to optimize cardiac filling and output without substantial hemodilution, which decreases peripheral vascular resistances and substantially alters blood conductivity. Small, but significant, increase in the dry-to-wet ratio of the heart and decrease in total hemoglobin take place as soon as 1 h of fluid support (Zuurbier et al., 2002).

The high metabolic rate and the high surface-to-volume ratio of rodents translate into a fast heat loss. Considering the significant impact of temperature on, for instance, cardiac output and ventricular pressures, it is crucial to maintain body temperature at 37 ± 1°C by monitoring and maintaining the animal temperature with a heating pad and a rectal probe, respectively, as well as minimizing body cavity opening during PV-loop analysis.

Although not mandatory, it is advisable to continuously measure ECG and peripheral oxygen saturation.

### Pressure-Volume Catheter Placing

Hemodynamic measurements using PV-loops can be assessed with a closed or open-chest approach. While in the former, the PV catheter is inserted by cannulating the right common carotid artery, the latter is accomplished through a sternotomy or thoracotomy and subsequent ventricular catheterization by an apical puncture, as described below.

Due to its lower invasiveness, the closed-chest approach is more suitable for long experiments such as acute drugs effects since the animals remain stable for a longer period. It is surgically more challenging, showing high probability of damaging the aortic valve when trying to insert the catheter down the aorta into the ventricle and imposing difficulties to correctly catheterize the ventricles and to perform load manipulations. Contrarily, an open-chest approach is recommended when hemodynamic evaluation is the terminal procedure or in animal models of aortic valve calcification, transverse aortic constriction-induced hypertrophy or atherosclerosis, specifically in the carotid artery. In the open-chest approach, after venous catheterization, a left lateral thoracotomy (rat) or a subxiphoid incision (mouse) is made to access the heart. To avoid bleeding during the thoracic incision, two haemostatic forceps may be used to clamp the tissue on each side of the incision. Before removing the haemostatic forceps, burn the bleeding tissues with thermal cautery. Carefully dissect the ascending or descending aorta until it is exposed enough to place a flow probe in rat or mouse, respectively. The flow probe allows assessing cardiac output. Next, puncture apically the left and/or right ventricle using a correct needle gauge with a diameter similar to the selected PV catheter. The adequate catheter should be selected according to the ventricle length so that, upon insertion along the longitudinal axis of the ventricle, the upper and lower (volume) electrodes should be positioned right next to the aortic valve and apical endocardial border, respectively. Only this positioning allows to accurately measure electric potentials proportional to the changes in ventricular volumes throughout the cardiac cycle (Wei et al., 2005). The pressure sensor is localized between these volume electrode pairs. A cotton swab helps to gently position the heart for the puncture.

### Animal Stabilization and Data Acquisition

**FIGURE 2:**
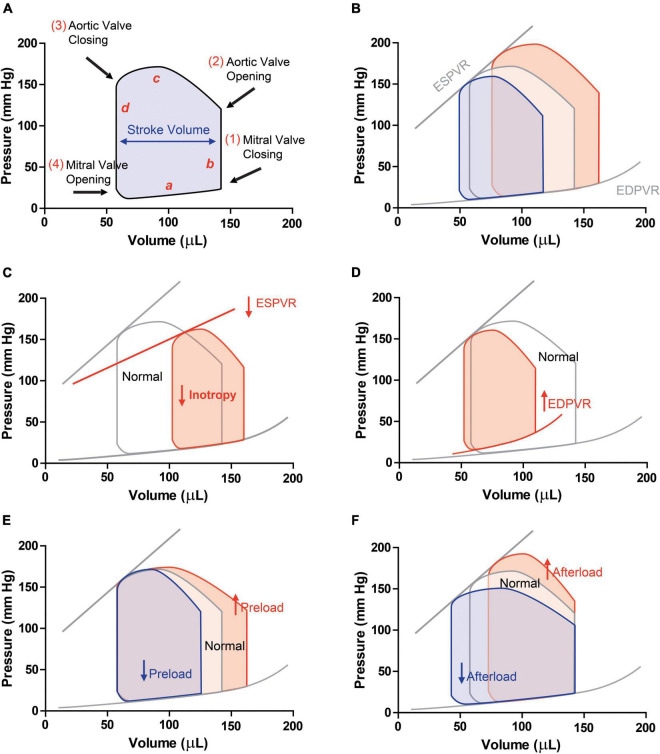
Pressure-volume loops in **(A)** physiologic conditions (a, ventricular filling; b, isovolumetric contraction; c, ejection; d, isovolumic relaxation). **(B)** PV loops with different preloads are obtained by occluding the vena cava. This manoeuvre allows obtaining end-diastolic pressure-volume relationship (ESPVR, slope of the linear regression) and end-diastolic pressure-volume relationship (EDPVR, slope of the exponential curve), load-independent indexes of contractility and stiffness, respectively. PV-loops in a condition of **(C)** systolic dysfunction, and HFrEF, in which ESPVR, SV and EF decrease and **(D)** diastolic dysfunction and HFpEF, in which hypertrophy and increased stiffness are observed. Schematic PV-loops drawing of increased (red) or decreased (blue) preload **(E)** and afterload **(F)**.

Before data acquisitions or any treatment, the position of the catheter should be correctly adjusted until the maximal value of stroke volume (SV) is achieved and a typical rectangular PV loop is visualized (Figure 2). To facilitate left ventricle catheter positioning, place the catheter inside the aorta (until an aortic trace is observed) and then, carefully pull the catheter until a good PV loop is obtained. Baseline and manipulation of loading conditions can be performed to guarantee that the electrodes are not in contact with intracardiac structures such as the papillary muscles. After stabilization of the signal, record baseline PV loops at steady-state and at variable preload or afterload conditions (vena cava occlusion or aortic constriction, respectively). Vena cava occlusions can be performed by placing a loop of suture around the vena cava below the level of the cardiac apex and pulling up on the suture for ∼6 heart beats to obstruct blood flow. Aortic constriction can be done with forceps after isolating the aorta and before placing the flow probe by suddenly clamping the aorta for 1–2 cycles. This procedure is used to derive various load-independent indices of systolic and diastolic function as described below. Between acquisitions allow pressure and volume to stabilize by returning to baseline values.

### Pressure and Volume Calibrations

Pressure-volume catheters have two pairs of upper and lower conductance electrodes (the outers are emitters and the inners are sensing electrodes) with a pressure-measuring sensor in between, providing simultaneous conductance and pressure signals (Pacher et al., 2008). Before ventricular catheterization, the pressure sensor typically undergoes a 2-point calibration via the software and/or transducer box, by which volts are converted to mmHg (usually 0, 25, or 100 mmHg) and subsequently the catheter offset value is determined by a zero-point calibration step by placing the catheter near the surface of a saline solution.

As PV catheter records conductance, it is necessary to convert the signal to volume. Volume calibration can be complex and requires a deep understanding of how the PV catheter works. The first calibration necessary to obtain accurate ventricular volumes is the conversion of volts into conductivity units (milliSiemens, mS) using a 2-point calibration via the software and/or transducer box, as mentioned above.

Secondly, one should carry out saline/conductance calibration. As PV catheters record conductance, it is necessary to exploit the relationship between electrical conductance and volume. A high-frequency low amplitude alternate current runs between these conductance electrodes to generate an electric field inside the heart. This electric field spreads out through the blood and surrounding myocardium, declining inversely to its distance to the electrodes. Considering a constant intensity emitted, the voltage between this pair of electrodes increases with the resistance of the surrounding fluid/structures (or decreases with their conductance) according to Ohm’s Law [Voltage (*V*) = Resistance (R) × Current (I)]. One should be aware that, besides the varying intraventricular blood volume, the conductance includes a quasi-constant signal from the myocardial ventricular walls. As conductance (G) is the inverse of resistance, the total conductance (G_x_) signal includes blood conductance (G_*b*_) and muscle or parallel conductance (G_*p*_). To estimate Gp, a small bolus of hypertonic saline solution (10–30% NaCl, <200 μl, or <20 μl for rat and mice, respectively) is rapidly infused spanning 5–8 beats, usually via the venous access for fluid support. This bolus transiently raises blood conductivity due to the increased concentration of electrolytes. Slow injections will be less effective in achieving this purpose. The relation between recorded conductance at end-diastole (G_ed_) and end-systole (G_es_) can be described as:


$$G_{\text{es}}=\left(m\times G_{\text{ed}}\right)+b$$

Where m is the slope and b is the intercept of the regression line. Parallel conductance (Gp), also called V_*p*_, can be estimated by finding the intersection between this regression and an identity line. This corresponds to the point where G_es_ and G_ed_ would theoretically be the same, i.e., for a theoretical empty ventricle. Therefore, this conductance is a reasonable surrogate of parallel conductance. By solving the equation (Ges = Ged) Gp = b/m.

After this calibration, the conductance signal of the blood (G_*b*_) can be easily converted to volumes by using the Baan’s equation:


$$\mathrm{Vi}\,\left(t\right)=\left(1/\alpha \right)\times L^{2}\times \sigma_{b}\times \left[G_{X}\,\left(t\right)-\mathrm{Gp}\right]$$

Where Vi (*t*) is the time-varying volume, α can be calculated as detailed below, L is the distance between the sensing electrodes, σ_*b*_ is the specific resistivity of the blood assessed by a rho cuvette that measures intrinsic resistivity of blood (see below), G_X_ is the total conductance measured, and G_*p*_ is the parallel (or muscle) conductance obtained upon hypertonic saline injection.

An alternative to the cuvette calibration is to compute the slope factor α, a correction factor that results from the ratio between the SV or the cardiac output (CO) assessed by the conductance catheter and the same parameters assessed by an independent method, such as magnetic resonance imaging (MRI), echocardiography or an aortic flow probe. In mice, it is reasonable to assume that α is very close to 1, since the cavity size is very small electrical field heterogeneity is not an issue. For rats, ideal calibration requires an aortic transit time flow probe which measures cardiac output in real-time and simultaneously to the PV loop acquisition (usually placed in the ascending aorta, it is also possible to place it in the descending aorta and estimate cardiac output on the assumption that 70% of cardiac output flows through the descending aorta). Using imaging method carried out before the PV loop evaluation is prone to bias due to different anesthetic conditions, changes in volume or hemodynamic changes induced by opening the thorax. Another alternative is to use a standard plexiglass cuvette with known volumes, which are filled with each animal heparinized blood.

The third calibration is the cuvette calibration that allows assessing the conductivity/resistivity of each animals blood. This circumvents the need to calibrate for blood resistivity and slope factor α. Upon subtracting G_*p*_, conductance values can be interconverted to volume by using the regression retrieved from the standard cuvettes. The most widely used and accepted method for volume calibration uses the mS values obtained after placing the conductance catheters in a plexiglass cuvette with known volumes, which are filled with each animal heparinized blood.

After converting measured conductance to volume signals using Baan’s equation, real-time PV relations can be obtained. However, this method has some fragilities since its precision is limited by the assumption of a linear conductance-volume relationship.

## Pressure-Volume-Loop Data Acquisition

The sampling rates of PV-loop data acquisition should be 5–10 times higher than the maximal expected heart rate. Low sampling rates lead to irreversible loss of information and to an inaccurate representation of the original signal. Too high sampling rates will create large files that increase data storage size and processing requirements of the coupled computer and may give rise to excessive noise. Most suppliers recommend default frequencies between 1 and 2 kHz. Amplification and range should be matched to double the highest expected signal amplitude.

Noise can be critical and sometimes it is only possible to solve the problem by applying filters to process your data. Filter settings should be used with moderation and consistently in all files from the same protocol and not only in the noisier ones. Low-pass filters are usually used to reduce noise, while high pass filters stabilize the baseline of a signal, for instance, minimizing the baseline drift in an ECG signal. The best approach is to reduce the noise by grounding of electrical equipment, which can come from nearby cables, other electric equipment interference or even a broken/dirty PV catheter. Noise problems are more frequent in volume channels. After a careful assessment of noise origin and correction, if the problem persists, the use of filters in volume channels signals should assure that the derived volume parameters are only minimally affected. The use of filters in pressure signals should be avoided as it may profoundly affect the derived parameters (e.g., +dP/dt).

While recording data, most data acquisition softwares allow inserting comments and annotations, which we recommend as a standard procedure as it facilitates the subsequent data analysis. Create and consistently apply internal controls and cut-off values; for instance, defining an algorithm that allows to quantitatively consider baseline hemodynamic values as stable or setting the lowest acceptable heart rate and end-systolic pressure for your experimental groups, which may be highly influenced by factors such as the depth of the anesthesia or the amount of blood loss.

After a period of stabilization, hemodynamic recordings should look like an ideal pressure-volume loop with a rectangular appearance (Figure 2).

## How to Analyze Pressure-Volume-Loops and Interpret the Derived Parameters

Analysis of PV-loops allows obtaining several derived-pressure and volume quantitative measurements that reports changes in cardiovascular and hemodynamic performance, some of them are not readily measurable by other methods (Table 2).

Figure 2A depicts the PV relationship for a single cardiac cycle. Each PV-loop is interpreted anticlockwise and divided into four phases: ventricular filling (a, diastole), isovolumic contraction (b, systole), ejection (c, systole), and isovolumic relaxation (d, diastole). Points 1 and 3 of the PV loop represent ventricular end-diastolic pressure (EDP) and end-diastolic volume (EDV) or end-systolic pressure (ESP) and end-systolic volume (ESV), respectively. The width of the loop represents the stroke volume (SV), resulting from the difference between EDV and ESV. The area within the loop is the ventricular stroke work (Table 1).

**TABLE 1:** PV loop derived parameters.

Parameter		S/D	Description
ESV	End-systolic volume	S	Ventricular volume at the end of systole
EDV	End-diastolic volume	D	Ventricular volume at the end of diastole
P_max_	Maximal pressure	S	Maximal pressure during systole
P_min_	Minimal pressure	D	Minimal pressure during diastole
ESP	End-systolic pressure	S	Pressure reached at the end of the systole
EDP	End-diastolic pressure	D	Pressure reached at the end of diastole
PE	Potential energy		Is defined by the area between the ESPVR and EDPVR curves to the left of the PV loop. PE = ESP (ESV−V_0_)/2−EDP (EDV−V_0_)/4, where V_0_ is the theoretical volume when no pressure is generated
PRSW	Preload recruited stroke work		Is calculated as the linear regression of stroke work with the end-diastolic volume. The slope of the PRSW relationship is a highly linear index of myocardial contractility that is load insensitive
PVA	Pressure volume area		Represents the total mechanical energy (TME) generated by ventricular contraction. This is equal to the sum of the stroke work (SW), encompassed within the PV loop, and the elastic potential energy (PE)
dP/dt_max_	Maximal velocity of pressure rise	S	Reported as maximal rate of pressure change in the ventricle. dP/dt_max_ are dependent on load and heart rate. LV dP/dt_max_ occurs before aortic valve closure
dP/dt_min_	Maximal velocity of pressure decrease	D	Reported as minimal rate of pressure change in the ventricle. dP/dt_min_ are dependent on load and heart rate. LV dP/dt_min_ is a marker of the start of left ventricular isovolumic relaxation
SV	Stroke volume	S	The volume ejected in each cardiac cycle The width of the PV-loop (Figure 1). SV = EDV−ESV
SW	Stroke work	S	The area of the PV-loop (Figure 1). SW = (EDV−ESV) × (ESP−EDP)
EF	Ejection fraction	S	Indicates the percentage change in LV volumes EF = SV/EDV × 100
HR	Heart rate		Number of cardiac cycles (beats) per minute
CO	Cardiac output	S	The amount of blood pumped by the ventricle by unit time. Is calculated as stroke volume multiplied by heart rate
CI	Cardiac index	S	CI = CO/body-surface-area (calculated as BW^^2/3^)
Ea	Arterial elastance		A measure of arterial load and its impact on the ventricle. Calculated as the simple ratio of ventricular end-systolic pressure to stroke volume
Tau	Exponential decay of the ventricular pressure during isovolumic relaxation	D	The time constant for ventricular pressure fall during active relaxation. Is the preferred method for assessing relaxation because it can be measured more accurately than dP/dt_min_, relaxation half time, and isovolumic relaxation time
CT	Contraction time	S	Duration of isovolumic contraction
RT	Relaxation time	D	Duration of isovolumic relaxation
ESPVR	End-systolic pressure-volume relationship	S	Represents the contractility of the ventricle. Describes the maximal pressure that can be developed by the ventricle at any given cardiac chamber volume. The slope and x-intercept of the ESPVR is generated experimentally by occluding the inferior vena cava (Figure 2)
EDPVR	End-diastolic pressure-volume relationship	D	Described the passive curve during ventricular filling and thus represent the passive proprieties of the myocardium during passive diastole. The EDPVR slope is reciprocal to ventricular compliance or stiffness. This curve is experimentally obtained after occlusion of inferior vena cava

*S, systole; D, diastole.*

**TABLE 2:** Main advantages and disadvantages of pressure-volume loop analysis to assess cardiac function.

Advantages of PV-loops	Disadvantages of PV-loops
Abundant and continuous data acquisition	Long-learning curve – surgical skills are needed
Load-independent measures of cardiac function	Invasive acute procedure
Pressure-derived parameters	Impossibility to perform longitudinal studies
High temporal resolution	Need to optimize fluid replacement
Reproductivity	Always requires anesthesia
Low maintenance cost	Open-chest approach requires mechanical ventilation
Free from radiation	Open-chest approach deviates from physiology

Pressure-volume analysis allows measuring ventricular function independently of the loading conditions and heart rate, representing a particular advantage of this technology over others techniques. Specific load-independent cardiac indexes of contractility include end-systolic pressure-volume relationship (ESPVR), dP/dt_max_-end-diastolic volume relation, maximal elastance (E_max_) and preload recruitable stroke work (PRSW). A load-independent measure of diastolic function is the end-diastolic pressure-volume relationship (EDPVR; Pacher et al., 2008). Two of these indexes will be briefly described below. For more information, please consult (Boron and Boulpaep, 2016).

For instance, the maximal pressure developed by the ventricle at any given ventricular volume is defined by the ESPVR, which represents the inotropic state of the ventricle. ESPVR is obtained experimentally upon occluding the inferior vena cava. This reduces venous return to the heart and decreases ventricular preload (EDV), causing the PV loop to shift to the left and become smaller over several cardiac cycles (reducing SV as assessed by the loop width, Figure 2B). Peak systolic pressure also decreases as the cardiac output declines during vena cava occlusion. The ESPVR is determined by the line intersecting the upper left corners of the loops (Figure 2B). When inotropy declines, the slope of ESPVR decreases (Figure 2C).

The slope of the passive filling curve (EDPVR) represents ventricular stiffness. For example, in ventricular hypertrophy, this slope is typically increased as the ventricle is stiffer. This results in higher filling pressures at any given ventricular volume (Figure 2D). Contrarily, when the ventricle undergoes eccentric remodeling (dilates), it becomes less stiff (more compliant) and therefore the slope of the filling curve decreases.

Ventricular PV-loops are an excellent tool for visualizing changes in ventricular function in response to changes in preload, afterload (Figures 2E,F), and inotropy. These ventricular changes can be complex because preload, afterload, and inotropy are interdependent variables, meaning that when one variable is changed, the other variables change. Therefore, it is of major importance to understand the independent effects of each of these variables on the ventricular function when the other variables are held constant.

To examine the independent effects of preload, assume that aortic systolic and diastolic pressure (afterload), and inotropy are held constant. Changes in preload affect the SV through the Frank-Starling mechanism. When venous return increases, preload increases and cardiac muscle fibers stretch as the ventricle fills to a greater extent (end-diastolic volume is increased; red loop in Figure 2E). With no change in afterload or inotropy, the ventricle will eject blood to the same end-systolic volume despite the increase in preload.

Afterload is the pressure (load) that the ventricle must generate to eject blood into the aorta. Changes in afterload affect the ability of the ventricle to eject blood and thereby alter stroke volume, end-systolic pressure and volume. Alteration in afterload results from changes in aortic and pulmonary vessel stiffness, wave reflection and small vessel resistance. Increased afterload will impact the duration of isovolumic contraction and the ventricle will need to generate a higher pressure to overcome, for instance, an elevated aortic diastolic pressure. Therefore, ejection begins at a higher aortic diastolic pressure. If preload (end-diastolic volume) and inotropy are held constant, this will result in a smaller stroke volume and an increase in end-systolic volume (red loop in Figure 2F).

Systolic dysfunction refers to impaired ventricular contraction (loss of inotropy) with a significant impact on SV and end-systolic volumes. Heart failure caused by systolic dysfunction is commonly referred to as heart failure with reduced ejection fraction (HFrEF). Under these conditions, the slope of the ESPVR decreases, leading to an increase in end-systolic volume (red loop in Figure 2C).

Diastolic dysfunction results from changes in ventricular diastolic properties that impact ventricular filling and/or relaxation. About 50% of heart failure patients have diastolic dysfunction, showing ejection fraction within the normal range (>50%). Heart failure caused by diastolic dysfunction is commonly referred to as heart failure with preserved ejection fraction (HFpEF). For instance, a reduction in ventricular compliance, increases the slope of EDPVR, compromises ventricular filling (decreased end-diastolic volume) and increases end-diastolic pressure (red loop in Figure 2D). Depending on the relative change in stroke volume and end-diastolic volume, there may or may not be a small decrease in ejection fraction.

Some PV-loop parameters are also influenced by which cardiac access is chosen. Open-chest model decreases venous return, therefore cardiac output falls. However, a comparation of open/close-chest model described that close-chest approach presents a larger ejection fraction and SV with a leftward shift in ventricular volume, lower end-systolic pressure and a more significant mismatch in the arterial-ventricular coupling (Lips et al., 2004).

During data analysis, experimenters should make consistently repeatable selections for analysis and data export. Uniform annotations and data acquisition procedures are key as current analysis software are not far from allowing a fully automatized analysis.

## Minimal Requirements for Pressure-Volume-Loop Analysis

A ventilator is mandatory for the open thorax method, and it should ideally allow adjusting pressure and volume ventilation parameters to properly inflate the lungs (inflation rates, respiratory rates, and positive-end-expiratory pressure).

Dramatic temperature fluctuations will have a major impact on cardiovascular function, thus, a heating pad with rectal temperature control probe is highly advisable to stabilize the animals’ temperature and hemodynamics.

Although not mandatory, a stereo microscope or any other magnifier might facilitate the visualization of small structures and vessels, especially in mice. Also, a vaporizer of halogenated gasses is recommended for a tighter anesthetic control and to minimize the cardio-depressant effects of other injectable anesthetic drugs.

If well maintained, fine and high-quality surgical instruments are a good long-term investment. A thermal cautery unit is of great utility to prevent excessive bleeding and a significant decrease in the circulating volume. Other useful disposable materials include gauze sponges, cotton-tipped applicators, sutures and tracheal cannula (24–24 G for mice and 14–16 G for rats, depending on their size).

A data acquisition system with an adequately high frequency of acquisition is essential for real-time visualization and data recording. Finally, several catheter suppliers commercialize transducers, amplifiers and PV catheters, which are essential for assessing ventricular pressures and volumes.

## Conductance Versus Admittance Catheters

The conductance catheter has a non-uniform electric field; thus, the relationship between conductance and volume is non-linear (Wei et al., 2005). In practice, the two major criticisms of this method are, on the one hand, the fact that the calibration factor α is incorrectly used in Baan’s equation as a constant despite the recognition that it changes dynamically as the moving heart wall changes the shape of the applied electric field. On the other hand, the assumption of G_*p*_ that as a constant value to be subtracted to total conductance despite its dynamic changes during the cardiac cycle. Parallel muscle conductance changes as the cardiac muscle moves closer to or farther away from the catheter (Porterfield et al., 1985).

To overcome this weakness, a time-varying G_*p*_ (admittance) and factor α (Wei’s equation), both instantaneously determined, were proposed as a solution that allows calculating dynamically the changes in G_*p*_ and α, respectively (Porterfield et al., 1985). By assuming a non-linear connection between conductance and volume (γ), Wei’s equation corrects for the non-homogeneous nature of the catheter electric field distribution and increases the accuracy of the conductance catheter system (Wei et al., 2005).


$$\text{Vi}\,\left(t\right)=1/\left(1-\frac{G\,b}{\gamma }\right)\,\rho \,L\,\wedge \,2\,\left(G\,b\right)$$

Where Vi (*t*) is the time-varying volume, ρ is the blood resistivity, L is the distance between the sensing electrodes, G_*b*_ is the blood conductance measured and γ is the field correction factor that is determined as:


$$\gamma =\frac{-b\pm \sqrt{b^{2}-4\,a\,c}}{2\,a}$$

a = SV−ρL^2^ (Gb_ED_−Gb_ES_)

b = −SV (Gb_ED_ + Gb_ES_)

c = SV × Gb_ED_ × Gb_ES_

The admittance technique relies on a measurable phase angle difference (θ) due to the presence of myocardium between the input current and the output voltage. In blood (alone) there is no measurable phase angle. However, both muscle and blood have a measurable electrical conductivity that affects the output signal magnitude. Admittance magnitude is the ratio of input current to output voltage (Porterfield et al., 1985). Measurement of both the magnitude and the phase of admittance allows estimation of the time-varying myocardial contribution, which provides a substantial improvement by eliminating the need for hypertonic saline injection (Raghavan et al., 2009). Thus, using admittance the contribution of the adjacent tissues is instantaneously removed with no need to conductance technique calibration with hypertonic saline injections or cuvettes.

## Advantages and Disadvantages of Hemodynamic-Derived Pressure-Volume-Loops

Both echocardiography and MRI allow for complete, repeated, and non-invasive assessment of cardiac structure as well as systolic and diastolic function. MRI shows the advantage of providing information regarding fibrosis. However, its use is limited due to the elevated costs. Contrarily, echocardiography is widely used and guidelines have been recently published aiming to increase accuracy and reproducibility between labs (Zacchigna et al., 2021). However, none of these methodologies allows assessing load-independent cardiovascular parameters that are not affected by cardiac preload or afterload. Indeed, PV-loops provide load-independent parameters allowing to differentiate if a certain contractility or relaxation variation derived from myocardial intrinsic changes or simply from the Frank-Starling effect (Pacher et al., 2008; Boron and Boulpaep, 2016). This is achieved by analyzing sequential PV-loops while reducing preload through experimental vena cava occlusion. On the one hand, the ESPVR, a contractility index, is given by the slope of the linear regression (Ees, end systolic elastance) obtained by plotting all ESP and ESV values. On the other hand, the end-diastolic pressure-volume relationship, a measure of stiffness, is given by the slope that best fits the exponential curve obtained by plotting all EDP and EDV values (Figure 2A). Also, PV-loops provides a significant amount of cardiac function and structure data that can be collected continuously and acquired during a pharmacological intervention. Other advantages and disadvantages of PV loops are depicted in Table 2.

## Other Applications of Pressure-Volume-Loops

Despite being the gold standard method to evaluate cardiac function in detail, the traditional hemodynamic analysis with conductance catheters has some shortcomings that inevitably impact cardiac function such as the need for anesthesia and animal manipulation. Furthermore, in chronic protocols, the progression of the disease or its recovery cannot be assessed over time. Telemetry systems provide an interesting alternative to overcome these limitations by allowing continuous and long-term data registries of multiple animals simultaneously. Indeed, since the 50’s that wireless technologies have been incorporated in implantable devices, which have been miniaturized in a feasible way to be used in rodent research (Nelson et al., 2020). Over the last two decades, an implantable telemetry system has been developed for measuring ventricular PV-loops in conscious, freely moving rats by connecting a small implantable bluetooth signal transmitter to a conductance catheter (Sato et al., 1994; Uemura et al., 2004).

The Baan equation is also applied in telemetry-derived PV-loops to convert conductance signals into volumes (Uemura et al., 2004). In addition to the conductance technique, admittance is another valid way to measure ventricular volumes. Some authors claim that admittance has advantages compared to conductance as discussed earlier in this article. Thus, in 2006, a PV-loop based on the measurement of real-time admittance was developed for hemodynamic measurements in conscious and ambulatory rats (Raghavan et al., 2008).

## Conclusion

The accuracy and precision of PV loop studies mainly depend on the suitable positioning and calibration of the catheters and the coupled instruments. Systematic procedures during experiments will significantly reduce the variability between individuals from the same group when it comes to data analyses, and thus, the number of animals required to demonstrate a physiologic, pathologic, or pharmacologic effect (3R’s policy). In addition, care should be taken to select which parameters are critical for the ongoing experimental design/protocol – under certain circumstances, relative changes might be enough.

Volume calibration is mandatory in all the experiments as it overcomes two main sources of error. First, the surrounding myocardium has conductivity. Second, the electric field produced by the catheter is not uniform and an independent measure of stroke volume (echocardiography or flow probe) should be used to adjust the volume signal. This is particularly critical for PV-loops derived from conductance catheters.

In conclusion, this article has described methods for optimizing the use of PV catheters for continuous monitoring of small rodents cardiac function. It also highlights how to detect, prevent and correct inaccurate volume and pressure measurements, ensuring optimal hemodynamic measurements and shortening the learning curve to properly use the PV catheter technology.

## Data Availability Statement

The original contributions presented in the study are included in the article/supplementary material, further inquiries can be directed to the corresponding author.

## Author Contributions

DM-S, IF-P, and VS wrote the manuscript. AL and IF-P reviewed the manuscript. All authors contributed to the article and approved the submitted version.

## Conflict of Interest

The authors declare that the research was conducted in the absence of any commercial or financial relationships that could be construed as a potential conflict of interest.

## Publisher’s Note

All claims expressed in this article are solely those of the authors and do not necessarily represent those of their affiliated organizations, or those of the publisher, the editors and the reviewers. Any product that may be evaluated in this article, or claim that may be made by its manufacturer, is not guaranteed or endorsed by the publisher.
